# Lodgepole pine, jack pine, and their hybrids: molecular markers reveal mountain pine beetle host-range expansion into jack pine of the boreal forest

**DOI:** 10.1186/1753-6561-5-S7-O3

**Published:** 2011-09-13

**Authors:** Catherine Cullingham, Sophie Dang, Corey Davis, Barry Cooke, David Coltman, Janice Cooke

**Affiliations:** 1 University of Alberta, Canada; 2 Northern Forestry Centre, Canadian Forest Service, Natural Resources Canada

## Background

**Figure 1:**
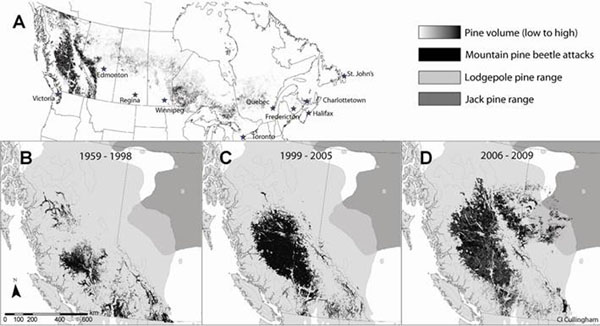
Canadian data for A. pine distribution, B. historical MPB range, C. extent of the current MPB outbreak prior to 2006, D. extent of the current MPB outbreak following dispersal into northern Alberta. MPB data are overlaid on classic lodgepole and jack pine ranges [1]. Pine volume and MPB attack data courtesy of the Canadian Forest Service.

Lodgepole pine (*Pinus contorta* Dougl. ex Loud. var. *latifolia*) is found in western North America, extending from the Yukon into British Columbia and Washington, and along the Rocky Mountains and eastern slopes to Colorado [1]. Jack pine (*Pinus banksiana* Lamb.) is a closely related species found east of the Rockies, mainly in Canada’s boreal forest from the Northwest Territories to Quebec [1]. Lodgepole and jack pine ranges overlap in northern Alberta and the Northwest Territories, where these species hybridize (Fig. 1). In the absence of reliable molecular markers, morphological characteristics are commonly used to distinguish lodgepole pine, jack pine and hybrids. However, hybrids and pure species can be difficult to visually distinguish, particularly at the presumed periphery of the hybrid zone, which is poorly described.

Mountain pine beetle (MPB; *Dendroctonus ponderosae* Hopkins) is indigenous to western North America. In the current MPB outbreak, more than 14 million hectares of mainly lodgepole pine forests have sustained MPB-caused mortality in British Columbia alone [2]. Following MPB long range dispersal into northwestern Alberta in 2006, MPB has continued its apparently unprecedented eastward spread into the lodgepole x jack pine hybrid zone of Alberta (Fig. 1).

To better define this hybrid zone, we developed microsatellite and SNP markers that distinguish the pure species from hybrids. We then used the microsatellite markers to test the hypothesis that the MPB epidemic has spread into jack pine.

## Materials and methods

Foliage was collected from 678 trees representing 25 localities in British Columbia, Alberta, Saskatchewan, Ontario, and Minnesota in 2007, 2008 and 2010, including 154 MPB-attacked trees from British Columbia and Alberta.

Eleven microsatellite markers amplifying both lodgepole and jack pine were developed from published loci [3] and loci isolated from a microsatellite enriched (GT_n_/CT_n_) lodgepole pine library. Genotyping and scoring was carried out as described [4]. There were less than 2% missing data, with a 0.8% genotyping error rate. Loci met Hardy-Weinburg equilibrium criteria; pairs of loci in linkage disequilibrium were negligible. Genetic diversity measures were calculated in GENEPOP [5], GenAlEx [6] and HP-RARE [7]. Assignment tests were conducted in NEWHYBRIDS [8] and STRUCTURE [9].

*In silico* species-discriminating SNP detection was carried out using CLC Genomics with lodgepole and jack pine cDNA 454 sequence data, and validated with PCR.

## Results and discussion

Genetic diversity was high among localities, and was higher in lodgepole than jack pine. Differentiation among localities (*F_ST_* = 0.125) and between species (*F_ST_* = 0.133) was high. Within species, differentiation among localities was generally low (*F_STlodgepole_* = 0.033, *F_STjack_* = 0.016).

The efficacy of the microsatellite loci to distinguish pure species and hybrids was tested by assignment of individuals to their correct class with NEWHYBRIDS and STRUCTURE, using five simulated datasets (ten iterations each) containing multiple levels of admixture generated with known pure lodgepole and jack pine as benchmarks. Using *Q_T_* greater than or equal to 0.9 to assign pure species and *Q_T_* less than 0.9 to designate hybrids, we achieved accurate resolution of first and second generation hybrids in simulated datasets using both NEWHYBRIDS and STRUCTURE, but encountered diminishing power with advanced generations. There was very high agreement between NEWHYBRIDS and STRUCTURE; a decision tree was developed to resolve the small number of disagreements. Assignment of the 678 genotyped individuals suggests that the intersection of the ranges classically described for lodgepole and jack pine [1] underestimates the distribution of hybrids. Pure lodgepole and jack pine were identified within the lodgepole x jack pine hybrid zone, with lodgepole pine often occupying higher elevations.

Eight of 154 MPB-attacked trees genotyped with the microsatellite loci were identified as jack pine. This first evidence of MPB mass-attack and reproduction on jack pine in natural stands was presented to key forest managers and decision makers via the National Forest Pest Strategy – a Canadian Forest Service initiative coordinating federal and provincial forest management and policy development – and led to accelerated development of a consensus inter-jurisdictional strategic plan.

We have developed 9 nuclear, 12 chloroplast, and 3 mitochondrial SNP markers that distinguish lodgepole pine, jack pine, and hybrids. These markers are being used to further delineate the hybrid zone, investigate gene flow and introgression, and continue monitoring MPB invasion of the boreal forest.

## Conclusion

Microsatellite markers reliably distinguishing lodgepole pine, jack pine and their hybrids were used to better delineate Alberta’s lodgepole pine x jack pine hybrid zone and to conclusively demonstrate that MPB has undergone host range expansion into jack pine of the boreal forest, a new habitat for this devastating forest pest.
